# Select gene mutations associated with survival outcomes in ER‐positive ERBB2‐negative early‐stage invasive breast cancer: A single‐institutional tissue bank study

**DOI:** 10.1002/cam4.70035

**Published:** 2024-07-19

**Authors:** Victor C. Kok, To‐Yu Huang, Yi‐Chiung Hsu, Yuan‐Ching Chang, Po‐Sheng Yang

**Affiliations:** ^1^ Division of Medical Oncology Kuang Tien General Hospital Cancer Center Taichung Taiwan; ^2^ Department of Medical Research MacKay Memorial Hospital New Taipei Taiwan; ^3^ Department of Biomedical Sciences and Engineering National Central University Taoyuan Taiwan; ^4^ Center for Astronautical Physics and Engineering National Central University Taoyuan Taiwan; ^5^ Department of General Surgery MacKay Memorial Hospital Taipei Taiwan; ^6^ Department of Medicine Mackay Medical College New Taipei Taiwan

**Keywords:** ER+/HER2‐, multigene mutational panel, prognostication, relapse‐free survival, targeted sequencing, tissue bank

## Abstract

**Introduction:**

The prognostic capability of targeted sequencing of primary tumors in patients with estrogen receptor‐positive, human epidermal growth factor receptor‐2‐negative early‐stage invasive breast cancer (EBC) in a real‐world setting is uncertain. Therefore, we aimed to determine the correlation between a 22‐gene mutational profile and long‐term survival outcomes in patients with ER+/ERBB2‐ EBC.

**Patients and Methods:**

A total of 73 women diagnosed with ER+/ERBB2‐ EBC between January 10, 2004, and June 2, 2008, were followed up until December 31, 2022. Univariate and multivariate Cox models were constructed to plot the relapse‐free survival (RFS) and overall survival (OS). The log‐rank test derived *p*‐value was obtained. For external validation, we performed a survival analysis of 1163 comparable patients retrieved from the Molecular Taxonomy of Breast Cancer International Consortium (METABRIC) dataset.

**Results:**

At follow‐up, 16 (21.9%) patients had relapsed, while 21 (nearly 29%) harbored mutant genes. Thirty‐three missense mutations were detected in 14 genes. The median ages were 51 and 46 years in patients with and without mutations, respectively. Patients with any mutation had a 1.85‐fold higher risk of relapse (hazard ratio [HR]: 1.85, 95% confidence interval [CI]: 0.60–5.69) compared to those without any mutation. Patients who harbored any of the six genes (*MAP2K4, FGFR3, APC, KIT, RB1*, and *PTEN*) had a nearly 6‐fold increase in the risk of relapse (HR: 5.82, 95% CI: 1.31–18.56; *p* = 0.0069). Multivariate Cox models revealed that the adjusted HR for RFS and OS were 6.67 (95% CI: 1.32–27.57) and 8.31 (*p* = 0.0443), respectively. METABRIC analysis also demonstrated a trend to significantly worse RFS (*p* = 0.0576) in the subcohort grouped by having a mutation in any of the six genes.

**Conclusions:**

Our single‐institution tissue bank study of Taiwanese women with ER+/ERBB2‐ EBC suggests that a novel combination of six gene mutations might have prognostic capability for survival outcomes.

## Abstract 2

We studied the prognosis of a 22‐gene mutational profile using tissue bank tumors of 73 women with ER+/ERBB2‐ EBC. This cohort of female patients had extended follow‐up, reaching 18.8 years. Any mutation in the MAP2K4, FGFR3, APC, KIT, RB1, and PTEN genes would have a 5.8‐fold increased risk of relapse.

## 1 INTRODUCTION

If current trends persist, the global burden of breast cancer is expected to increase, with the annual incidence of newly diagnosed patients projected to reach approximately 3.2 million worldwide by 2040.^1^ This anticipated 40% increase in incidence holds true across nations regardless of their Human Development Index.^1^ Among the various subtypes of early‐stage invasive breast cancer (EBC), estrogen receptor‐positive, ERBB2‐negative (ER+/ERBB2‐) breast cancer accounts for approximately 60% of cases.^2^ Unlike other breast cancer subtypes, such as triple‐negative and HER2‐enriched breast cancer, which commonly relapse within the first few years of follow‐up, ER+/ERBB2‐ invasive breast cancer often presents with a late recurrence, manifesting 5–10 years or longer after primary treatment.^3^

The prediction of late recurrence is based on specific clinicopathological characteristics including tumor size, lymph node involvement, tumor grade, and the level of Ki‐67 proliferation.^4^ However, a recent study aimed to identify the significant prognostic factors of luminal B‐like/ERBB2‐negative EBC in women reported that traditional clinicopathological factors such as age, menopausal status, tumor size, progesterone receptor status, overall histological grade, and Ki‐67 index were not significantly associated with subsequent relapse.^5^

Over the past two decades, the number of studies utilizing genomic information, such as somatic mutational profiles, for prognostication in breast cancer has increased significantly. Leveraging targeted sequencing of primary breast carcinoma to optimize prognostication in early breast cancer, by adding molecular mutational profiles to the current clinicopathological determinants, is a prominent area of ongoing research. For example, three recently published studies collectively emphasize the importance of integrating molecular mutational profiles with existing clinicopathological determinants to improve prognostication in early breast cancer.^6^, ^7^, ^8^ In these studies, key genomic alterations such as *PIK3CA*, *TP53*, *FGFR1*, and *MAP3K1* mutations play significant roles in predicting disease outcomes and guiding treatment decisions.

However, gene expression assays are not yet widely adopted for prognostication purposes in patients with ER+/ERBB2‐ EBC. Thus, the potential of multigene mutational panel testing to analyze specific cancer‐related gene hotspots using next‐generation sequencing (NGS) for prognostication in this group remains largely unexplored. In addition, whether the somatic mutational profile of primary breast cancer impacts the prognosis of patients with ER+/ERBB2‐ EBC remains unknown.

The emergence of efficient and cost‐effective sequencing technology offers an opportunity to assess specimens stored in tissue banks using multigene panel NGS, even decades after the initial diagnosis. Consequently, this study aimed to assess the prognostic value of a 22‐gene mutational profile obtained via targeted sequencing of primary breast cancer specimens from patients with ER+/ERBB2‐ EBC archived in the tissue bank under an extended follow‐up.

## 2 MATERIALS AND METHODS

This cross‐platform study involved the diagnosis, treatment, and follow‐up of accrued patients, tissue bank specimen procurement and storage, NGS, and cBioPortal dataset analysis. This study was approved by the Institutional Review Boards (IRBs) of MacKay Memorial Hospital (12MMHIS121) and Academia Sinica (AS‐IRB02‐102086). All patients provided informed consent for using their data and samples in research.

### 2.1 Patient population and tissue bank

Between January 10, 2004, and June 2, 2008, 73 consecutive women with ER+/ERBB2‐ nonmetastatic EBC provided their primary tumor specimens to the MacKay Memorial Hospital Tissue Bank. We restaged the tumors according to the Union for International Cancer Control/American Joint Committee on Cancer (AJCC) 7th edition after its release in late 2011. Patients were treated according to the Clinical Practice Guidelines (CPG) and conformed to the National Comprehensive Cancer Network CPG. After obtaining IRB approval, tissue samples were retrieved from the tissue bank for NGS analysis.

### 2.2 NGS methodology

We previously^9^ described the method for NGS. Briefly, specific genomic DNA regions were enriched using a microdroplet‐based technique via a hotspot cancer panel (RainDance Technologies). A proprietary pipeline was used to design the primer library for this study. The functional point mutations included 54 missense mutations in 22 cancer‐related genes (Table S1). This library, when merged dropwise with sheared genomic DNA samples on the RainDance ThunderStorm™ Panel, resulted in a microdroplet‐based polymerase chain reaction on these genes, targeting over 13,000 known mutations from the Catalog of Somatic Mutations in Cancer database. The enriched samples were subsequently sequenced on the MiSeq platform using Illumina, producing 250‐bp paired‐end reads. The raw data were deposited under the BioProject ID PRJNA758602 in the National Center for Biotechnology Information Sequence Read Archive. Alignment of the reads against the University of California Santa Cruz Hg19 human reference genome was accomplished using BWA version 7.5a, enabling the identification of SNPs and indels. Variant annotation was obtained by combining the outputs from GATK software (v2.7–2) and SAMtools (v0.1.19) with the ANNOVAR package. The average sequencing depth was 5222‐fold, ranging from 2900 to 8633‐fold.

For a deeper understanding of gene functionality, we used the Ingenuity Pathway Analysis tool from QIAGEN. Further, we validated the results using Sanger sequencing on a 3500xL DX Genetic Analyzer (Life Technologies Co.) to ensure the accuracy of our findings, especially concerning APC mutations.

### 2.3 Patient‐level data extraction

Patient demographic data, including date of birth, menopausal status, date of diagnosis, 7th edition AJCC stage, date of last follow‐up, relapse, and death from cancer specific or other causes, were extracted. The treatment details included primary‐site surgery, adjuvant endocrine therapy, radiotherapy, and adjuvant chemotherapy. Pathological details, including histotypes (invasive carcinoma of no special type (ductal), invasive lobular carcinoma, and mucinous carcinoma), overall tumor grade, tumor size, nodal involvement, and tumors, were retrospectively assigned to either luminal A‐ or B‐like and luminal B‐like subtypes using tumor grade, ER, PgR, HER2 immunohistochemistry, or fluorescent in situ hybridization. In 2004 and 2008, the Ki‐67 index was not standardized at our institution, thus, the multigene Oncotype Dx testing was not applicable for this cohort of patients.

### 2.4 Selection of Six Candidate Genes for Exploratory Analysis

We selected three oncogenes (*MAP2K4*, *FGFR3*, *KIT*) and three tumor suppressor genes (*APC*, *retinoblastoma tumor suppressor gene* (*RB1*, *PTEN*) based on their involvement in the phosphatidylinositol‐3‐kinase (PI3K) pathway or their significance in univariate survival analysis (Table S2; Figure S3). The ultimate selection of the candidate genes was based on their involvement in signaling pathways such as PI3K, MAPK, RTK‐RAS, WNT, DNA damage response and repair, and cell cycle regulation. Although the individual effects of these genes were not statistically significant for prognostication, when considered together in an integrated manner, *MAP2K4* and *FGFR3* may serve as pivotal points connecting the PI3K signaling pathway with other pathways critical for the proliferation and survival of ER+ breast cancer tumors.^10^ Variant calling and its allele frequency of the six select genes of the primary tumors are presented in Table S4. We employed NGS to identify and analyze somatic mutations in six genes critically linked to cancer development within primary tumors. To ensure the validity of our findings, benign variants were excluded by comparing their frequencies with data from the Taiwan Biobank and The Cancer Genome Atlas database. This comparison focused on validating high‐frequency loss‐of‐function mutations. Our comprehensive analysis strategy accurately reflects the functional impacts of these mutations, emphasizing their significant roles in cancer pathogenesis. In addition, a filtering threshold of 1% was applied. Our lockdown data and statistical modeling were based on the filtered NGS results.

### 2.5 Validation of the select six mutated genes in the METABRIC dataset

We retrieved the mutational profiles of 1163 women with ER+/ERBB2‐ EBC from the Molecular Taxonomy of Breast Cancer International Consortium (METABRIC) dataset^11^ using the cBioPortal platform for Cancer Genomics.^12^ The METABRIC trial collected patient characteristics, breast tumor expression data, CNV profiles, and SNP genotypes. The patient selection criteria were similar to those of our cohort. Relapse‐free survival (RFS) was compared between patients in the altered and unaltered groups using the log‐rank test. Survival analysis was performed using the ER+/HER2‐negative EBC data retrieved from the METABRIC dataset. The RFS curves for 320 months (26.7 years) were plotted.

### 2.6 Statistics and software

The patients were divided into two subcohorts according to the mutation detected. Univariate and multivariate Cox proportional hazard model‐derived hazard ratios (HRs) with the corresponding 95% confidence intervals (CIs) were calculated to compare the RFS and overall survival (OS) rates. The Nelson–Aalen cumulative hazard was used to compare the cumulative relapse rate between the two groups. Taking all considerations with a two‐sided 5% type I error, the sample size of 73 patients will have 93.7% power to detect an effect size by the hazard ratio rate of 4.0 for a two‐sample comparison of survival functions by the select six‐gene signature. Statistical analyses were performed using GraphPad Prism software version 10.0.2 (GraphPad Software, La Jolla, CA, USA). The STATA 18 software (StataCorp 2023, Stata Statistical Software: Release 18, College Station, TX: StataCorp LLC) was used to plot the Nelson–Aalen cumulative hazards. Descriptive statistics of clinical and pathological variables are reported using frequencies for categorical variables or medians with minimum/maximum frequencies for continuous variables. Group‐wise comparisons of categorical data were evaluated using the chi‐squared test with two‐tailed *p*‐values generated using Fisher's exact test. Unpaired *t*‐tests and Mann–Whitney *U*‐tests were used to analyze the intergroup differences between age subgroups and follow‐up times. A *p*‐value <0.05 was considered statistically significant. For univariate survival analysis, Kaplan–Meier survival curves for OS and RFS were plotted and compared using the log‐rank test with reported *p*‐values. Patients without endpoint events at the last follow‐up visit were censored. In the unadjusted baseline setting, the assumption of proportional hazards was validated using the Schoenfeld residual plot and the log‐minus‐log survival plot (Figure S1). For multivariate prognosis, multiple Cox proportional hazards regression models were constructed based on the clinical and molecular variables identified as being significantly associated with survival in the univariate analysis. The corresponding effects of multivariate adjustments for clinical variables on the HRs, 95% CIs, and *p*‐values of the baseline model of the six genes were evaluated. The Bonferroni correction was applied for multiple comparisons of RFSs in patient subgroups with or without a mutated gene of interest (Table S6).

## 3 RESULTS

In total, 73 women were diagnosed with ER+/ERBB2‐ EBC between January 10, 2004, and June 2, 2008, and followed up prospectively until December 31, 2022. The longest follow‐up reached 18.8 years (median = 163.2 months), during which 16 patients relapsed. Twenty‐one of the 73 (28.8%) patients had tumors with at least one mutation. A total of 33 missense mutations were detected in 14 genes. ESR1 mutations were not assessed in the 22‐gene panel because resistance to aromatase inhibitors in an adjuvant setting was not considered. Patients were grouped according to whether their primary breast tumor harbored a mutation in any of the 14 genes or not (*n* = 21; *n* = 52, respectively) (Table 1). The median age was 48 years; the youngest patient was aged 29 years old, while the oldest patient was aged 84 years old. Premenopausal, perimenopausal, and postmenopausal women accounted for 34%, 29%, and 37% of the cohort, respectively. Invasive lobular and mucinous carcinomas accounted for 7% and 4% of the cohort, respectively; other patients had invasive carcinomas of no special type. According to stage distribution, 34% of the patients had stage I, 51% had stage 2, and 15% had stage III tumors. Approximately 18% of the tumors that demonstrated grade III characteristics or PgR negativity were classified as luminal B‐like tumors.^13^ Figure S2 shows the distribution of the mutated genes according to the number of somatic mutations in one specimen and each gene of interest. One patient harbored four synchronous mutations: *PIK3CA, TP53, RB1*, and *KIT*. One patient had three mutations: *PIK3CA*, *TP53*, and *MSH2*. Seven patients had two mutations, while 12 patients had only one mutation.

**TABLE 1:** Demographic data distribution and prospective survival outcomes of the cohort of women with estrogen receptor‐positive, human epidermal growth factor receptor‐2‐negative early‐stage invasive breast cancer whose primary tumors were examined by targeted sequencing for mutations.

	Entire cohort (*n* = 73)	Patients without mutation (*n* = 52)	Patients with any mutation (*n* = 21)	*p*‐Value
Age, median (min‐max)	48 (29–84)	46 (29–78)	51 (36–84)	0.0444
Menopausal state				0.1057
Pre‐menopausal	25 (34%)	21 (40%)	4 (19%)	
Peri‐menopausal	21 (29%)	15 (29%)	6 (29%)	
Post‐menopausal	27 (37%)	16 (31%)	11 (52%)	
Histotype				0.4248
Invasive carcinoma of no special type (ductal)	65 (89%)	45 (87%)	20 (95%)	
Lobular carcinoma	5 (7%)	5 (10%)	0 (0%)	
Mucinous	3 (4%)	2 (4%)	1 (5%)	
Tumor grade, overall				0.3585
Grade I	16 (22%)	13 (25%)	3 (14%)	
Grade II	48 (70%)	34 (65%)	17 (81%)	
Grade III	3 (4%)	2 (4%)	1 (5%)	
Missing	3 (4%)	3 (6%)	0 (0%)	
Stage by AJCC ver. 7th				0.0268
Stage I	25 (34%)	22 (42%)	3 (14%)	
Stage II	37 (51%)	25 (48%)	12 (57%)	
Stage III	11 (15%)	5 (10%)	6 (29%)	
Luminal A‐like or B‐like^a^	60 (82%)	43 (83%)	17 (81%)	*p* > 0.9999
Luminal B‐like^a^	13 (18%)	9 (17%)	4 (19%)	
Follow‐up time, median (min, max)	163.2 (3.8, 225.9)	178.4 (6.0, 225.9)	152.4 (3.8, 219.0)	0.1269
Relapse‐free survival (mo.), median (95% CI)	142.0 (84.0–182.0)	151.5 (83.0–190.0)	106.0 (59.0–182.0)	0.2829
Overall survival (mo.), median (95% CI)	163.0 (106.0–190.0)	178.5 (121.0–195.0)	152.0 (60.0–189.0)	0.8436

^a^ Categorizing luminal type into A‐like or B‐like was due to a lack of Ki‐67 index data in the cohort initially diagnosed between January 10, 2004, and June 28, 2008.

Abbreviations: AJCC, American Joint Commission of Cancer; CI, confidence interval; mo., months.

**FIGURE 1:**
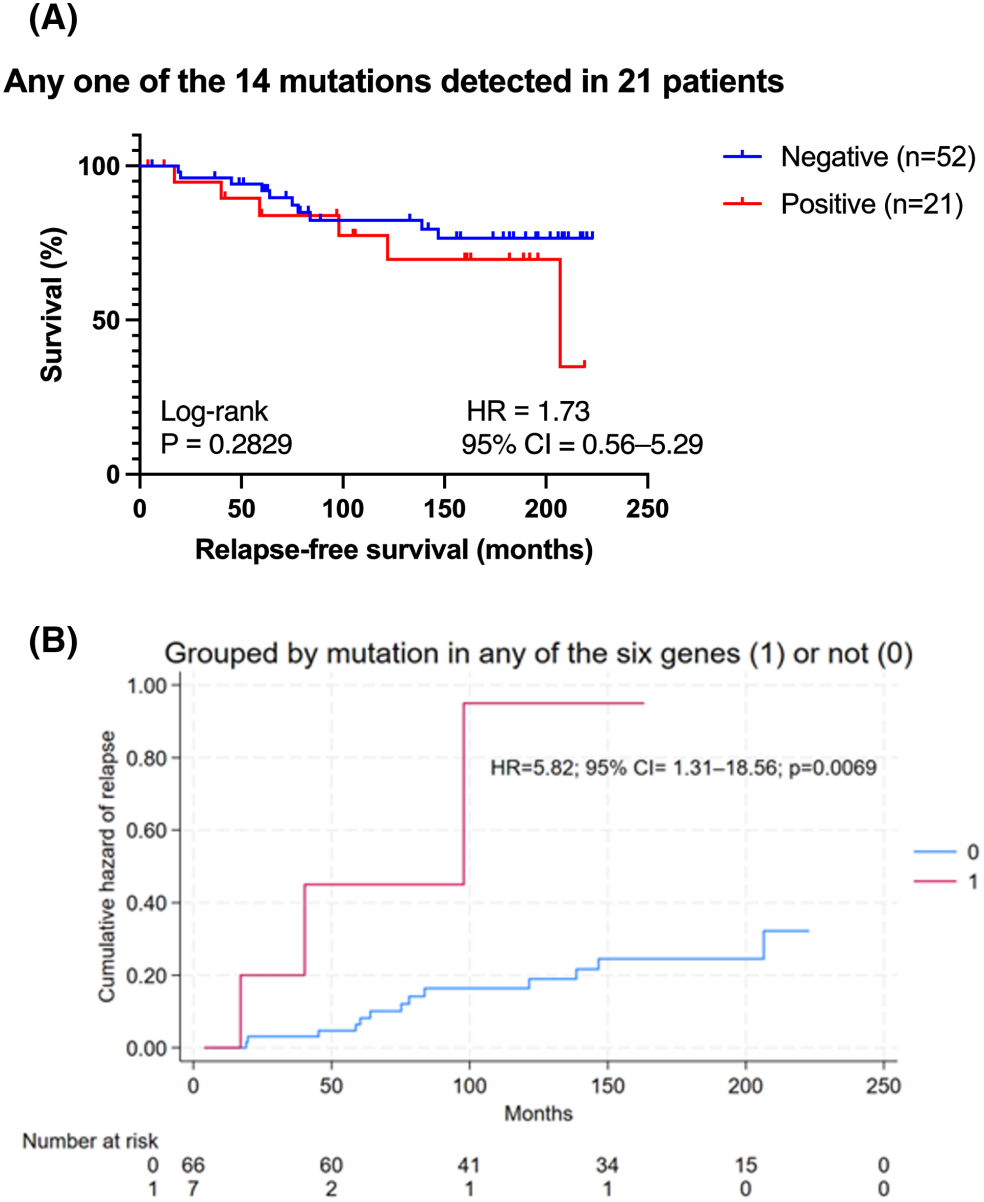
Comparison of relapse‐free survival (RFS) between the two subcohorts of women with ER+/ERBB2‐ early‐stage invasive breast cancer grouped based on the presence of specific mutation(s) in genes of interest. (A) Patients with tumors harboring any of the 14 mutations (*n* = 21) had a nonsignificant 73% increased risk of relapse. (B) Patients with tumors harboring any mutation in the six genes (*MAP2K4*, *FGFR3*, *APC*, *KIT*, *RB1*, and *PTEN*) (*n* = 7) had a 5.8‐fold increased risk of relapse compared with those who did not have mutations in the six genes (*n* = 66). The graph was derived from the Nelson–Aalen cumulative hazard function.

**FIGURE 2:**
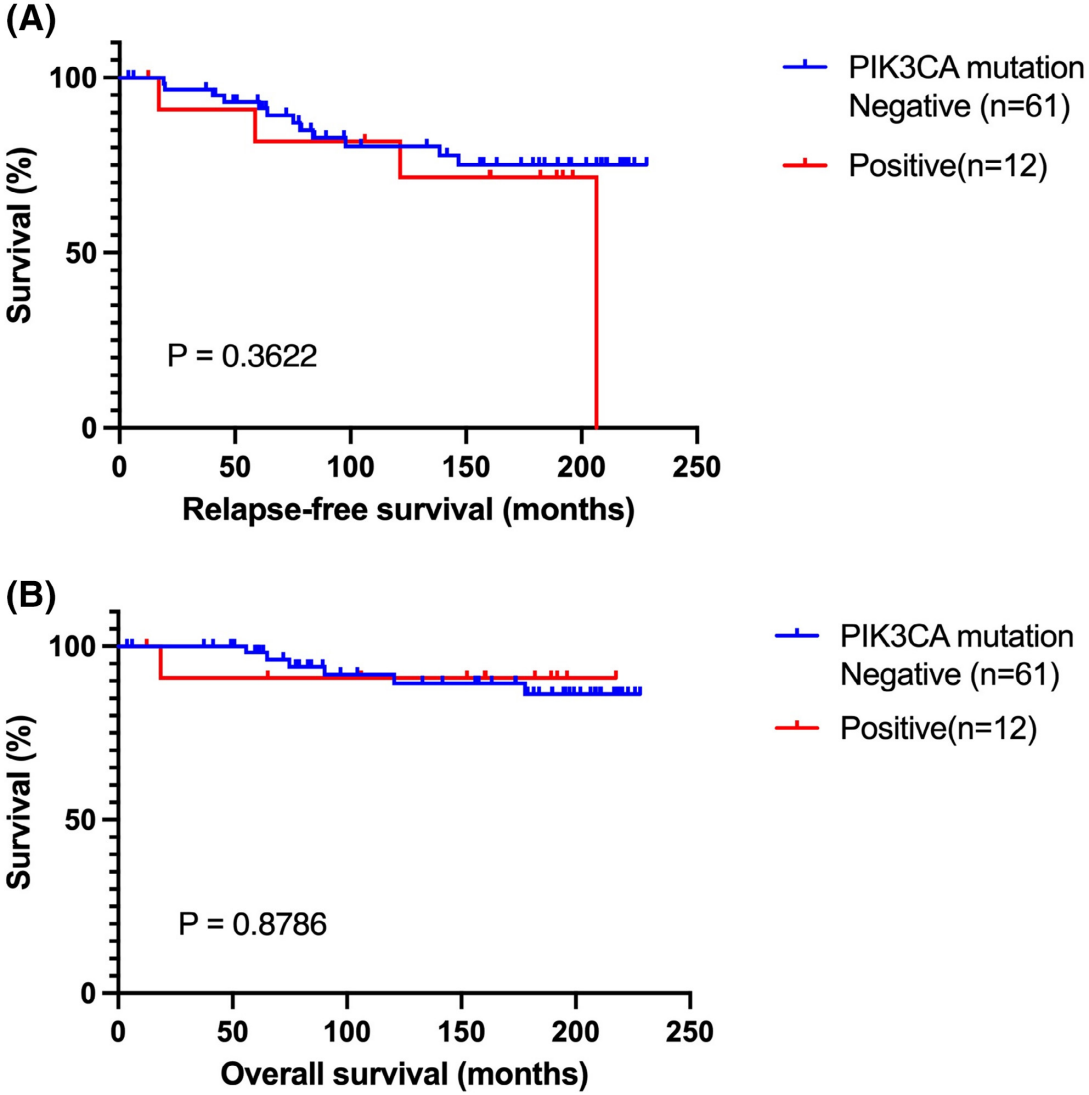
Both relapse‐free survival (RFS) and overall survival (OS) based on the presence (*n* = 12) or absence (*n* = 61) of PIK3CA mutation showed no impact on the prognosis of women with ER+/ERBB2‐ early‐stage invasive breast cancer. (A) RFS graph. (B) OS graph.

The RFS for the entire cohort was 142 months (95% CI: 84.0–182.0 months). Patients with any mutation in the 14 genes had a nonsignificant 73% increase in the HR (1.73, 95% CI: 0.56─5.29, Log‐rank *p* = 0.2829) with a numerically worse RFS of 106 months (95% CI: 59.0─182.0 months) compared to those without any mutation (RFS: 151.5 months, 95% CI: 83.0─190.0 months) (Figure 1 and Table 1). When grouped by mutation in any of the six genes, *FGFR3, RB1, APC, KIT, PTEN*, and *MAP2K4*, the unadjusted HR was 5.82 (95% CI: 1.31─18.56) with a *p* = 0.0069 (Table 2). The median PFS durations were 40.0 months for women with mutations in any of the six genes and 157.0 months for women who did not have mutations in any of the six genes (Table 2). The Nelson–Aalen cumulative hazard for relapse between the groups with or without mutations in any of the six genes is presented in Figure 2B. The adjusted HR was 6.67 (95% CI: 1.32–27.57; *p* = 0.0111) when the multivariate Cox model was adjusted for age, menopausal state, histotype, tumor grade, overall stage, and the retrospective intrinsic subtype approximation.

**TABLE 2:** Multivariate Cox proportional hazard model‐derived hazard ratio and 95% confidence interval (CI) comparing the relapse‐free survival and overall survival in women whose primary breast cancer harbored mutations in any of the six genes (*FGFR3, RB1, APC, KIT, PTEN*, and *MAP2K4*) compared with patients who did not harbor mutations in any of those genes.

	ANY MUTATION months, median	NO MUTATION months, median	Hazard Ratio	95% CI	*p* Value
Relapse‐Free Survival					
Univariate	40.0	157.0	5.82	1.31–18.56	0.0069
Model 1			5.60	1.21–19.30	0.0118
Model 2			6.67	1.32–27.57	0.0111
Overall Survival					
Univariate	42.0	176.0	9.07	1.29–42.70	0.0090
Model 1			9.95	1.17–68.53	0.0200
Model 2			8.31	0.90–???	0.0443

Model 1, Multivariate adjustment for age and stage, as seen in Table 1. Model 2, multivariate adjustment for age, menopausal status, histotype, tumor grade, stage by AJCC, and intrinsic subtype approximation.

??? means the upper limit of the 95% CI cannot be calculated.

The OS of the entire cohort was 163.0 months (95% CI: 106.0–190.0 months). Patients with tumors harboring any of the somatic mutations had numerically worse OS than patients with wild‐type tumors (152.0 months [95% CI: 60.0–189.0 months] vs. 178.5 months [95% CI: 121.0–195.0 months]) (Table 1). However, patients with any mutation in the highlighted six genes harbored a 9‐fold increased risk of death with an unadjusted HR of 9.07 (95% CI: 1.29─42.70; *p* = 0.0090) (Table 2). Multivariate Cox model 2 showed an adjusted HR of 8.31 (*p* = 0.0443) (Table 2).

*PIK3CA* mutation was the most common somatic mutation in the entire cohort, accounting for approximately 20% (12/73) of all cases (Figure 2). However, this gene did not show prognostic capability. Nevertheless, the *RB1* mutation had a detrimental effect on both RFS and OS, as shown in Figure S3A,B. Moreover, patients with APC mutation had a much poorer RFS of 39.69 months, with an adjusted HR of 40.60 (95% CI: 1.52–1.105; *p* = 0.0114) than patients without APC mutation (Table S3 and Figure S3E).

**FIGURE 3:**
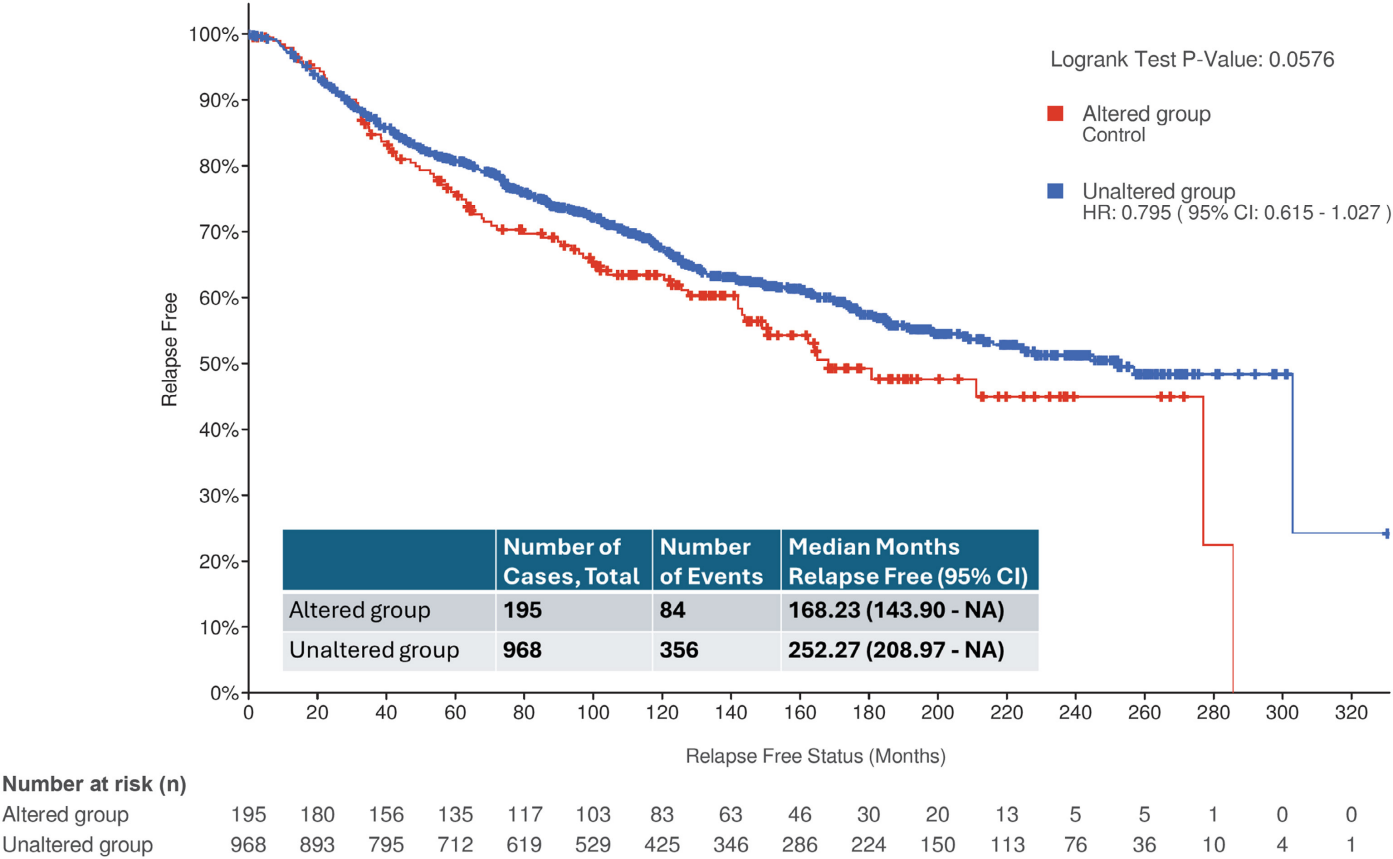
Comparison of relapse‐free survival (RFS) between the two groups of women with ER+/ERBB2‐ early‐stage invasive breast cancer with or without genetic alteration in the six genes (*MAP2K4*, *FGFR3*, *APC*, *KIT*, *RB1*, and *PTEN*). Patient data were from the METABRIC dataset assessable on the cBioPortal platform. Somatic mutation was the commonly detected genetic alteration. The altered group comprised 195 patients, while the unaltered group comprised 968 patients. With up to 320 months of follow‐up, the altered group had a numerically poorer median RFS (168.23 months) compared with the unaltered group (257.27 months). The unaltered group has an hazard ratio of 0.795 (95% CI, 0.615─1.027) with a trend to significance (*p*‐value = 0.0576).

In the METABRIC dataset, a total of 1163 patients had ER+/ERBB2‐ EBC. Among them, 195 (16.8%) patients had primary tumors with at least one mutation in the six genes. The frequencies and types of genetic alterations in these six genes were *MAP2K4* (4% mutation), *FGFR3* (0.5% copy number amplification), *APC* (2.1% mutation), *KIT* (0.3% copy number amplification), *RB1* (1.7% mutation), and *PTEN* (4.3% mutation). Patients in the altered group had a much worse RFS (168.23 months, 95% CI: 143.90—not reached) than those in the unaltered group (252.27 months, 95% CI: 208.97—not reached) with a trend to significance (*p*‐value = 0.0576) (Figure 3).

## 4 DISCUSSION

This study addresses the imperative need for improved prognosis in patients with ER+/ERBB2‐ EBC. Despite significant advancements in breast cancer research, the ability to predict survival outcomes accurately using clinically available targeted sequencing of primary tissues in real‐world settings remains a subject of ongoing investigation. In this context, our study sought to investigate the prognostication capability of a 22‐gene mutational profile obtained through targeted sequencing in women with ER+/ERBB2‐ EBC, whose primary breast cancer specimens were retrievable from a tissue bank. Our findings shed light on the potential effect of specific gene mutations on the long‐term prognosis of these patients.

Our study reveals critical insights into the potential role of specific gene mutations in predicting long‐term outcomes for patients with ER+/ERBB2‐ EBC. Notably, we identified six specific gene mutations, namely *MAP2K4, FGFR3, APC, KIT, RB1*, and *PTEN*, which were strongly associated with a significantly higher risk of relapse and poorer overall survival. The HRs for RFS and OS associated with these mutations surpassed those previously reported for this breast cancer subtype, suggesting the substantial prognostic capability of this six‐gene panel. Moreover, the median progression‐free survival (PFS) was markedly shortened by 84 months with a trend to statistical significance using the survival data of 1163 comparable patients from the METABRIC database.

However, we did not delve into the mechanistic aspects of why these specific gene mutations were associated with significantly poorer long‐term outcomes. Nonetheless, insights can be gleaned from basic scientific research which has provided a plethora of evidence on the mechanistic possibilities of the adverse impact of these select mutant genes on patients' long‐term outcomes. Three of the six genes (*MAP2K4*, *FGFR3*, *PTEN*) have been known to play a vital role in phosphoinositide 3‐kinase/Akt/phosphatase and tensin homolog/mammalian target of rapamycin (PI3K/Akt/PTEN/mTOR) signaling.^14^, ^15^, ^16^ *FGFR3* is reportedly involved in tamoxifen and fulvestrant resistance.^15^ *RB1* is a cell cycle regulator, implicated in coordinating multiple pathways leading to the disease progression of ER+ breast cancer and is related to the CDK4/6 activity—RB dependency integrated signature.^17^ *KIT* is the master regulator controlling the switching to a permissible scenario for tumor proliferation.^18^ Lastly, the *APC* alteration causes resistance to doxorubicin when administered in the neoadjuvant or adjuvant setting.^19^ Moreover, alterations, including mutation, in these genes might contribute to more aggressive disease behavior and reduced survival in patients with ER+/ERBB2‐ EBC. In addition, alterations in *MAP2K4*, *FGFR3*, and *APC* genes have been linked to cancer progression and treatment.^14^, ^20^, ^21^, ^22^ These cell‐line studies suggest that these genetic alterations drive more aggressive disease behavior, contributing to an elevated risk of relapse and reduced survival.

Our study had several strengths that enhance the significance of our findings. First, the extended follow‐up period spanning almost two decades provides a comprehensive view of the long‐term outcomes of patients with ER+/ERBB2‐ EBC, a subgroup known for late recurrences. This extended follow‐up, in conjunction with tissue bank‐derived somatic mutational profiles, contributes to the uniqueness of our research. Additionally, using archived primary breast cancer specimens from a tissue bank enriches the retrospective analysis, making it ambispective by combining both retrospective patient data and prospective NGS testing results.

Compared with previous studies,^23^, ^24^, ^25^ our findings indicate a substantial enhancement in the prognostic capability of specific gene mutations. Notably, the HRs for RFS and OS associated with these mutations were significantly higher than those previously reported.^24^ These selected somatic mutational sequencing results highlight the potential add‐on benefits of mutational profiling to conventional prognostic factors in this specific patient population. In the context of the current literature, the potential of multigene mutational panel testing for prognostic capability in patients with ER +/ERBB2‐ EBC remains largely unexplored. Previous studies have predominantly focused on examining the gene expression profiles to determine breast cancer prognosis.^26^, ^27^, ^28^, ^29^, ^30^ Our research bridges this gap by applying widely available targeted sequencing to select cancer‐related genes and shows promise in identifying high‐risk patients who may benefit from more intensive surveillance or targeted interventions.

Nevertheless, our study had some limitations. The relatively small sample size may limit the generalizability of our findings. Additionally, our study only focused on specific gene mutations. Other genetic alterations such as copy number variations can also play a significant role in prognosis. Moreover, selection bias may also be present as our analysis relied on archived tissue bank specimens despite our consecutive patient approach. Due to the study's retrospective nature and limited number of patients, the potential impact on the point estimate of the hazard ratio from treatment variation and adjuvant drug compliance issues cannot be fully controlled in the multivariate analyses, even though oncologists achieved consensus on institutional treatment guidelines. Therefore, large‐scale studies with diverse patient populations are warranted to validate the prognostic significance of these six gene mutations. In vitro and in vivo experiments are required to elucidate the mechanistic underpinnings of these mutations in breast cancer prognosis. Exploring the potential therapeutic implications of targeting these mutations is a valuable avenue for future research.

There has been substantial discourse in the literature on applying genetic mutations, whether germline or somatic, to enhance personalized treatment strategies.^6^, ^7^, ^8^, ^31^, ^32^ While our study provides novel insights into the genetic mutations associated with relapse and survival in ER‐positive, ERBB2‐negative EBC, translating these findings into clinical practice is not straightforward. The identification of specific gene mutations, such as MAP2K4, FGFR3, APC, KIT, RB1, and PTEN, which are linked to higher relapse risks and show a trend toward significance in the large METABRIC cohort, underscores the importance of further research before these genetic markers can be used to enhance patient stratification and personalized treatment strategies. By integrating these genetic insights into routine clinical assessments through targeted or comprehensive genome sequencing, oncologists can consider this novel combination to better identify patients at higher risk of relapse, thereby optimizing follow‐up schedules. This approach not only aims to improve patient outcomes but also to refine treatment plans based on individual genetic profiles, paving the way for more precise and effective management of breast cancer.

## 5 CONCLUSIONS

Our single‐institution tissue bank study of Taiwanese women with ER+/ERBB2‐ EBC suggests that we should leverage the mutational profile of the primary tumor for prognostic capability and discover valid somatic mutation signatures that can predict long‐term outcomes. Our six‐gene hypothesis calls for further investigation to examine its role in the prognostic capability and the potential impact on long‐term outcomes. Meanwhile, identifying these mutations in clinical practice should allocate more attention and resources from the care team to refine patient risk stratification and therapeutic strategies. Targeted sequencing of primary tissues in a real‐world setting offers exciting prospects for improving patient care.

## AUTHOR CONTRIBUTIONS

**Victor C. Kok:** Conceptualization (lead); formal analysis (lead); investigation (lead); methodology (lead); supervision (equal); validation (lead); visualization (lead); writing – original draft (lead); writing – review and editing (lead). **To‐Yu Huang:** Formal analysis (supporting); investigation (equal); methodology (equal); visualization (supporting); writing – review and editing (supporting). **Yi‐Chiung Hsu:** Data curation (lead); formal analysis (lead); investigation (lead); methodology (lead); resources (equal); software (lead); validation (equal); visualization (equal); writing – review and editing (equal). **Yuan‐Ching Chang:** Investigation (equal); validation (equal); writing – review and editing (equal). **Po‐Sheng Yang:** Conceptualization (equal); data curation (equal); formal analysis (equal); funding acquisition (equal); project administration (equal); resources (equal); supervision (equal); validation (equal); visualization (equal); writing – review and editing (equal).

## FUNDING INFORMATION

This study was supported by grants from the National Science and Technology Council, Taiwan (NSTC 112‐2321‐B‐195‐001 and 112‐2314‐B‐008‐003) and MacKay Memorial Hospital (MMH‐106‐73 and MMH‐107‐49), Taipei, Taiwan.

## ETHICS STATEMENT

This study was approved by the Institutional Review Boards (IRBs) of MacKay Memorial Hospital (12MMHIS121) and Academia Sinica (AS‐IRB02‐102086). All patients provided informed consent for future research when they donated their tumor specimens to the tissue bank. They also provided informed consent for using their data and samples in research.

## Supporting information


Figure S1.

Figure S2.

Figure S3.

Table S1.

Table S2.

Table S3.

Table S4.

Table S5.

Table S6.

## Data Availability

According to the Taiwan Personal Data Protection Act, the tissue bank research data cannot be made public.
