# Supplementary figures and images for: Select gene mutations associated with survival outcomes in ER‐positive ERBB2‐negative early‐stage invasive breast cancer: A single‐institutional tissue bank study
Source: Cancer Med. 2024 Jul 19;13(14):e70035. doi: 10.1002/cam4.70035 (PMC11258552; doi:10.1002/cam4.70035)

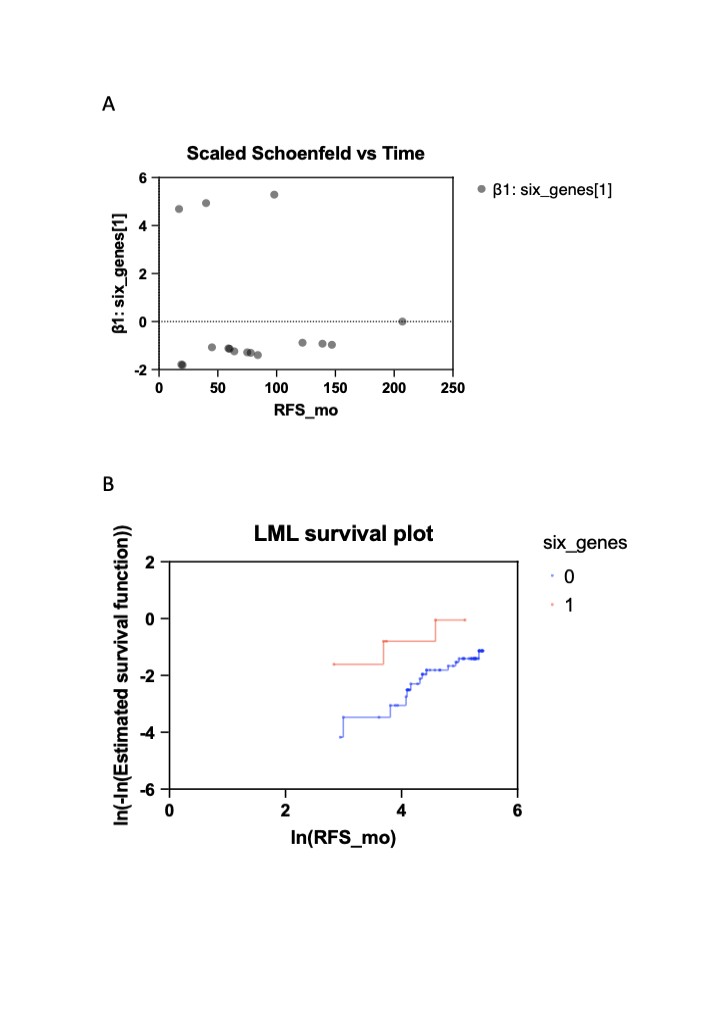

Supplement: Supplementary file 1 — Figure S1. [file CAM4-13-e70035-s003.jpg]

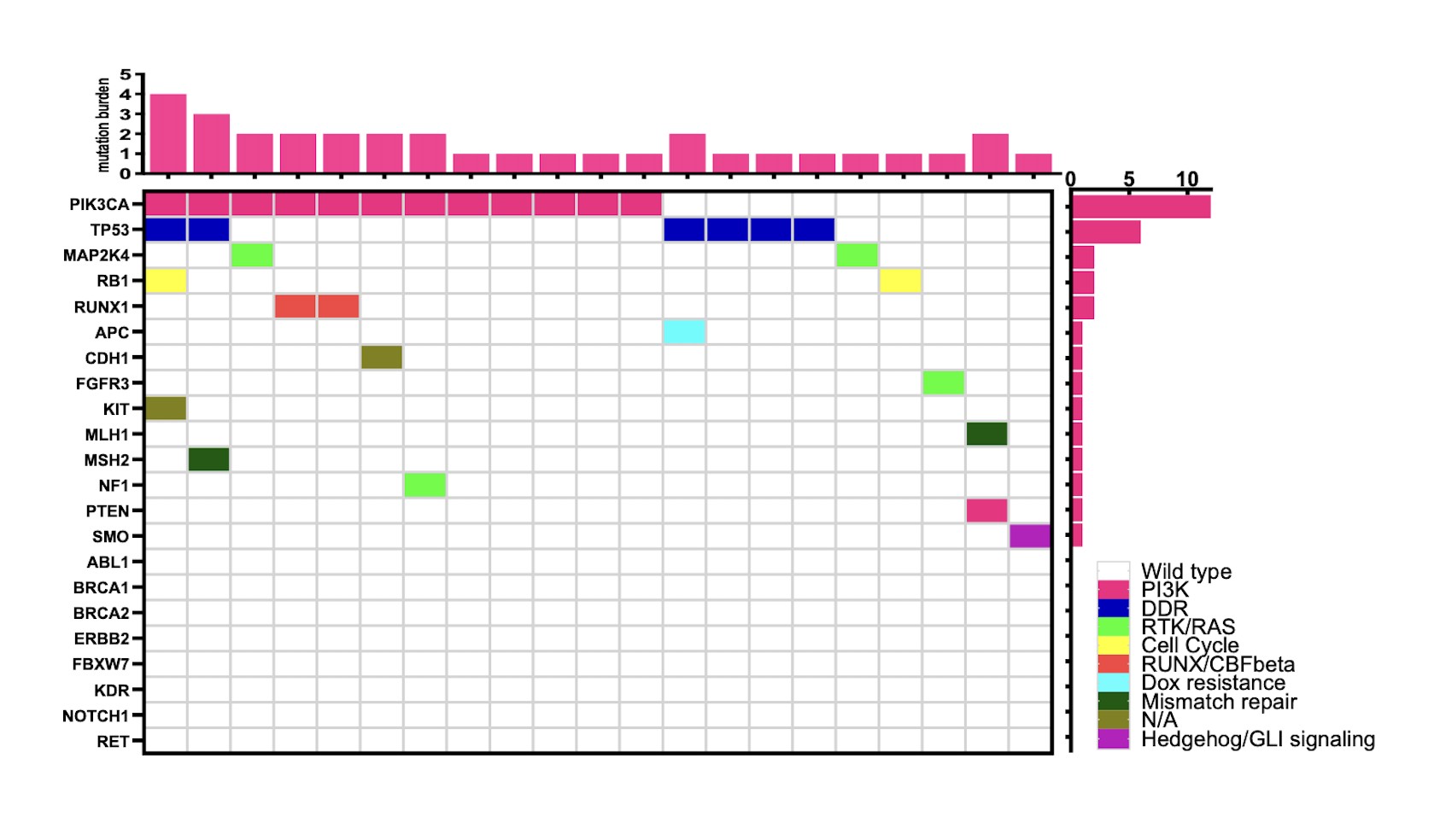

Supplement: Supplementary file 2 — Figure S2. [file CAM4-13-e70035-s001.jpg]

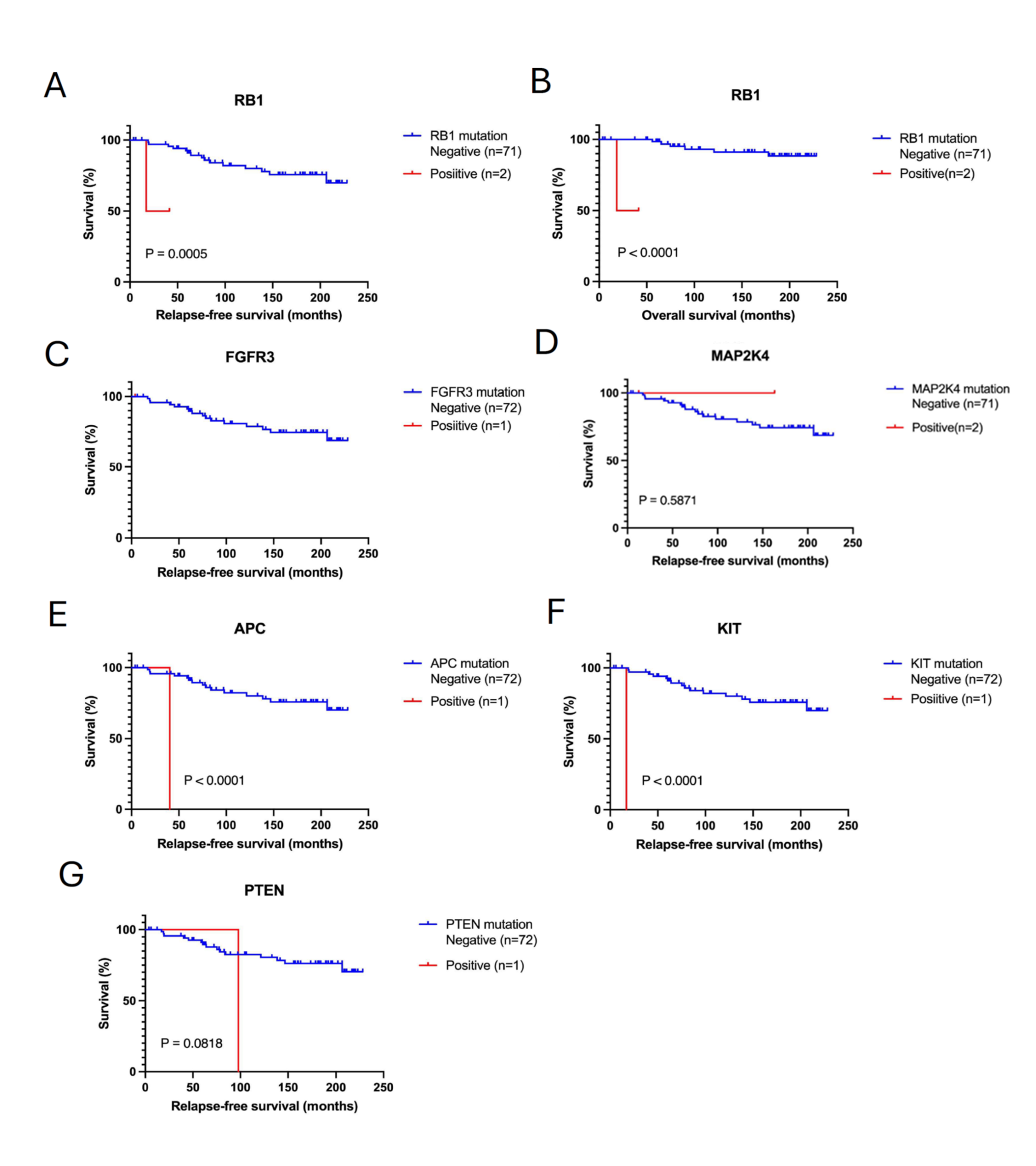

Supplement: Supplementary file 3 — Figure S3. [file CAM4-13-e70035-s006.tif]
