# Supplementary material for: Select gene mutations associated with survival outcomes in ER‐positive ERBB2‐negative early‐stage invasive breast cancer: A single‐institutional tissue bank study
Source: Cancer Med. 2024 Jul 19;13(14):e70035. doi: 10.1002/cam4.70035 (PMC11258552; doi:10.1002/cam4.70035)
Supplement: Supplementary file 4 — Table S1. [file CAM4-13-e70035-s002.docx]

| **Supplementary Table 1. List of specific signaling pathways and allocation of genes studied in the current study.** | | |
| --- | --- | --- |
| Pathway | Current Study | Ref in PMID |
| Homologous Recombination Deficiency | BRCA1, BRCA2, ABL1, KDR, TP53 | 36604691 |
| DNA Damage Response and Repair (DDR) | BRCA1, BRCA2, PTEN, TP53 | 36854505, 36655350 |
| PI3K | PIK3CA, PTEN | 32252811 |
| Cell Cycle | RB1 | 30511015 |
| TP53 | TP53 | 35173185 |
| RTK-RAS | ERBB2, NF1, KIT, FGFR3 | 37501682, 32821750 |
| MAPK pathway | MAP2K4 | 29795445, 22722193 |
| NOTCH | FBXW7, NOTCH1 | 36045911, 21134077 |
| EMT | ABL1 | 35719027 |
| WNT and doxorubicin-resistance | APC | 37108784 |
| Mismatch repair-deficient HR+ breast ca | MLH1, MSH2 | 34001143 |
| RUNX/CBFβ function | RUNX1 | 36831308 |
| Hedgehog-GLI signaling | SMO | 36674836 |
| Estrogen receptor pathway | RET | 35582269, 34824841 |
| Epithelial polarization and differentiation | CDH1 | 35348974 |
| 16 genes represent the most mutated genes in breast cancer. CDH1, GATA3, MAP2K4, MAP3K1, PTEN, AKT1, BRAF, ERBB2, ESR1, KIT, KRAS, PIK3CA, PIK3R1, RUNX1, SF3B1, TP53. (Ref.: Elena Lopez-Knowles et al. Molecular characterization of aromatase inhibitor-resistant advanced breast cancer: the phenotypic effect of ESR1 mutations. ***BJC*** 2019;120:247–255. | | |
