# Supplementary material for: Select gene mutations associated with survival outcomes in ER‐positive ERBB2‐negative early‐stage invasive breast cancer: A single‐institutional tissue bank study
Source: Cancer Med. 2024 Jul 19;13(14):e70035. doi: 10.1002/cam4.70035 (PMC11258552; doi:10.1002/cam4.70035)
Supplement: Supplementary file 5 — Table S2. [file CAM4-13-e70035-s009.docx]

| Supplementary Table 2. Details of the six mutated genes in the context of ER+/ERBB2- breast cancer (BC). | | | | |
| --- | --- | --- | --- | --- |
| **Gene** | **Oncogene or tumor suppressor** | **Frequency of somatic mutation in ER+/ERBB2- BC based on METABRIC** | **Involved Signaling Pathways** | **Prior research on ER+/ERBB2- BC (PMID-8 digits number)** |
| *MAP2K4* | OG | 4% | PI3K/AKT pathway. | Studied in non-specific type BC. (31761784) |
| *FGFR3* | OG | 0% or not done. (Copy number amplification 0.5%) | MAPK, PI3K/AKT, STAT, and PLCγ pathways. | Involved in tamoxifen and fulvestrant resistance (21792889). Implicated in inflammatory BC. (30086764) |
| *KIT* | OG | 0% or not done (Copy number amplification 0.3%) | The master regulator controls the switching to a permissible scenario for cell proliferation. | Studied in general. (32793804; 23582602; 29852185) |
| *APC* | TSG | 2.1% | Inactivated causes doxorubicin resistance. | (10854222) |
| *RB1* | TSG | 1.7% | Cell cycle regulator. | Related to the CDK4/6 activity-RB dependency integrated signature (32242058) |
| *PTEN* | TSG | 4.3% | PI3K/PTEN/mTOR pathway. Cell cycle regulator. | (37344170) |
