# Supplementary material for: Select gene mutations associated with survival outcomes in ER‐positive ERBB2‐negative early‐stage invasive breast cancer: A single‐institutional tissue bank study
Source: Cancer Med. 2024 Jul 19;13(14):e70035. doi: 10.1002/cam4.70035 (PMC11258552; doi:10.1002/cam4.70035)
Supplement: Supplementary file 6 — Table S3. [file CAM4-13-e70035-s007.docx]

| Supplementary Table 3. Unadjusted and multivariate Cox model-derived hazard ratios for relapse in women with estrogen receptor-positive, ERBB2-negative early-stage breast cancer whose primary tumor harbored an *APC* mutation. | | | |
| --- | --- | --- | --- |
| **Cox Model** | **Median Relapse-Free Survival (months)** | **Hazard ratio (95% Confidence Interval)** | ***P* value** |
| Unadjusted | 39.69 | 66.45 (2.63–1679) | 0.0030 |
| Controlling for age |  | 31.58 (1.21–832.8) | 0.0167 |
| Controlling for age and stage |  | 40.60 (1.52–1105) | 0.0114 |
