# Supplementary material for: Select gene mutations associated with survival outcomes in ER‐positive ERBB2‐negative early‐stage invasive breast cancer: A single‐institutional tissue bank study
Source: Cancer Med. 2024 Jul 19;13(14):e70035. doi: 10.1002/cam4.70035 (PMC11258552; doi:10.1002/cam4.70035)
Supplement: Supplementary file 7 — Table S4. [file CAM4-13-e70035-s004.docx]

**Supplementary Table 4.** Variant calling and its allele frequency revealed by next-generation sequencing in the six select genes of the primary tumors.

| *Gene* | Amino Acid Mutation(s) | Allele Frequency (%) |
| --- | --- | --- |
| *MAP2K4* | p.V192V | 31.30% |
|  | c.550_551T | 8.80% |
|  | p.T183I | 5.40% |
| *FGFR3* | p.T539T | 100.00% |
|  | p.T330T | 6.80% |
|  | p.T338M | 3.40% |
|  | NA (intronic) | 3.40% |
|  | p.P684P | 1.40% |
| *APC* | p.G1660G | 90.50% |
|  | p.T1475T | 89.80% |
|  | p.Y468Y | 81.00% |
|  | p.V1107A | 2.70% |
|  | p.T478T | 2.00% |
|  | p.Y201Y | 1.40% |
| *KIT* | p.K546K | 8.80% |
|  | p.L862L | 7.50% |
|  | p.M541L | 6.10% |
| *RB1* | NA (intronic) | 100.00% |
|  | NA (intronic) | 100.00% |
|  | NA (intronic) | 42.90% |
| *PTEN* | NA (intronic) | 66.70% |
|  | NA (intronic) | 12.20% |
|  | NA (intronic) | 1.40% |
| Footnote:  We employed next-generation sequencing to identify and analyze somatic mutations in six genes critically linked to cancer development within primary tumors. To ensure the validity of our findings, benign variants were excluded by comparing their frequencies with data from the Taiwan Biobank and The Cancer Genome Atlas database. This comparison focused on validating high-frequency loss-of-function mutations. Our comprehensive analysis strategy accurately reflects the functional impacts of these mutations, emphasizing their significant roles in cancer pathogenesis. In addition, a filtering threshold of 1% was applied. Our lockdown data and statistical modeling were based on the filtered NGS results. | | |
