# Supplementary material for: Select gene mutations associated with survival outcomes in ER‐positive ERBB2‐negative early‐stage invasive breast cancer: A single‐institutional tissue bank study
Source: Cancer Med. 2024 Jul 19;13(14):e70035. doi: 10.1002/cam4.70035 (PMC11258552; doi:10.1002/cam4.70035)
Supplement: Supplementary file 8 — Table S5. [file CAM4-13-e70035-s005.docx]

**Supplementary Table 5.** Sensitivity Analysis Assessing the Impact on Relapse-Free Survival (RFS) and Overall Survival (OS) by Adding *PIK3CA* to the Select Six Genes

|  | Select Six Genes | Adding *PIK3CA* |
| --- | --- | --- |
| RFS | | |
| Adjusted hazard ratio* (95% CI) | 6.67 (1.32─27.57) | 3.98 (1.12─14.21) |
| p-value | 0.0111 | 0.0298 |
|  | | |
| OS | | |
| Adjusted hazard ratio* (95% CI) | 8.31 (0.90─?) | 1.79 (0.19─13.16) |
| p-value | 0.0443 | 0.5783 |

*Multivariate adjustment for age, menopausal status, histotype, tumor grade, stage by AJCC 7^th^ edition, and intrinsic subtype approximation (same as that presented in Table 2).
