# Supplementary material for: Select gene mutations associated with survival outcomes in ER‐positive ERBB2‐negative early‐stage invasive breast cancer: A single‐institutional tissue bank study
Source: Cancer Med. 2024 Jul 19;13(14):e70035. doi: 10.1002/cam4.70035 (PMC11258552; doi:10.1002/cam4.70035)
Supplement: Supplementary file 9 — Table S6. [file CAM4-13-e70035-s008.docx]

| **Supplementary Table 6**. The Bonferroni correction for multiple comparisons of relapse-free survivals in patient subgroups with or without a mutated gene of interest. | | | |
| --- | --- | --- | --- |
| Pair comparison in a Cox model | Alpha level set at 0.05 | Bonferroni-adjusted new alpha level | Statistical significance |
| *PIK3CA* | *P* = 0.3622 | α_New_ = 0.05/7  = 0.007 | - |
| *RB1* | ***P* = 0.0005** |  | Significant! |
| *PTEN* | *P* = 0.0818 |  | - |
| *APC* | ***P* < 0.0001** |  | Significant! |
| *KIT* | ***P* < 0.0001** |  | Significant! |
| *FGFR3* | - |  | - |
| *MAP2K4* | *P* = 0.5871 |  | - |

**Boldface** means the P-value is significant.

RB1, PTEN, or KIT mutation was significantly associated with poor prognosis in each Cox model. Even after the Bonferroni correction for multiple pair-wise comparisons, the association remained statistically significant under a new alpha level.
